# Proceedings of the Fifth Annual Meeting of the Association of Medical Superintendents of American Institutions for the Insane

**Published:** 1850-10

**Authors:** 


*Proceedings of the Fifth Annual Meeting of the Association of Medical Superintendents of American Institutions for the Insane.*

The Association of Medical Superintendents of American Institutions for the Insane, convened at the Tremont House in the city of Boston, on the 18th day of June, 1850, at 10 o'clock, A. M.; the President, Dr. WILLIAM M. AWL, in the chair, and Dr. KIRKBRIDE, Secretary.

Present, Dr. *James Bates*, of the Maine Insane Hospital, Augusta.

Dr. *Andrew McFarland*, of the New Hampshire State Asylum, at Concord.

Dr. *William H. Rockwell*, of the Vermont Asylum for the Insane, Brattleboro, Vt.

Dr. *Luther V. Bell*, of the McLean Asylum for the Insane, at Summerville, Mass.

Dr. *C. H. Stedman*, of the Boston Lunatic Hospital.

Dr. *Edward Jarvis*, of the Dorchester (Mass.) Private Asylum.

Dr. *George Chandler*, of the Massachusetts State Lunatic Hospital, at Worcester.

Dr. *N. Cutter*, of the Pepperill (Mass.) Private Institution.

Dr. *Isaac Ray*, of the Butler Hospital for the Insane, at Providence, R. I.

Dr. *John S. Butler*, of the Connecticut Retreat for the Insane, at Hartford.

Dr. *N. D. Benedict*, of the New York State Lunatic Asylum, at Utica.

Dr. *C. H. Nichols*, of the Bloomingdale Asylum for the Insane, New York.

Dr. M. A. Ranney, of the New York City Lunatic Asylum, on Blackwell's Island.

Dr. *Henry W. Buel*, of Sandford Hall, (Private Institution,) Flushing, N. Y.

Dr. *H. A. Buttolph*, of the New Jersey State Lunatic Asylum, at Trenton.

Dr. *Thomas S. Kirkbride*, of the Pennsylvania Hospital for the Insane, at Philadelphia.

Dr. *J. H. Worthington*, of the Friends Asylum for the Insane, at Frankford, Pa.

Dr. *William S. Haines*, of the Philadelphia Lunatic Hospital, Blockley.

Dr. *John Fonerden*, of the Maryland Hospital for the Insane, at Baltimore.

Dr. *John M. Galt*, of the Eastern Asylum of Virginia, at Williamsburg.

Dr. *William M. Awl*, of the Ohio Lunatic Asylum, at Columbus.

Dr. *S. Hanbury Smith*, of the Ohio Lunatic Asylum, at Columbus.

Dr. R. J. *Patterson*, of the Indiana Hospital for the Insane, at Indianapolis.

Dr. *J. M. Higgins*, of the Illinois Hospital for the Insane, at Jacksonville.

Dr. *Edward Mead*, of the Chicago Private Retreat for the Insane, (Ill.)

The minutes of the last meeting having been read, the President announced in a feeling and appropriate address, the death of three members of the Association since its last meeting: Dr. Samuel B. Woodward, the first President of the Association, and formerly Superintendent of the Massachusetts State Lunatic Hospital,—Dr. Amariah Brigham, Superintendent of the New York State Lunatic Asylum, and Vice President of the Association,—and Dr. McNairy, Superintendent of the Tennessee Hospital for the Insane.

The Secretary reported that, as instructed by the Association, he had invited the Boards of Trustees or Managers of all the institutions for the insane, in the United States and British Provinces, to attend its meetings, and had received letters in reply from the Boards of Managers of the Maine Insane Hospital, Massachusetts General Hospital, Boston Lunatic Hospital, Friends Asylum, Pa., Maryland Hospital and Eastern Asylum of Virginia. On motion of Dr. Bates, it was

*Resolved,* That each member of the Association be authorized to invite such gentlemen to attend its sessions as he may deem proper.

Dr. Bell stated that in consequence of a full and well-written notice of the life and professional labors of our late associate, Dr. James Macdonald, of N. Y., having appeared in the American Journal of Insanity, he would suggest the adoption of that notice, instead of preparing another, specially for the use of the Association, which was approved.

The President stated that in obedience to the instructions of the Association, he had, soon after the last meeting, selected a subject for a report for each member, to all of whom due notice had been given, and from most of whom he had received answers accepting the duties assigned them.

An invitation from the Board of Trustees of the Boston Lunatic Hospital, to visit that institution, to-morrow at 4½ P. M., was read and accepted.

On motion of Dr. Bell it was

*Resolved,* That in order to enable the members of the Association, while performing the regular business that may come before the meeting, so to arrange their sessions as most satisfactorily to apportion their time, and be able to enjoy the hospitality that may be extended to them,—a business committee be appointed, who shall at the commencement of each morning session, report the papers to be read, and other matters to be attended to during the day. Drs. Bell, Bates, and Kirkbride, were appointed the committee.

On motion of Dr. Rockwell, it was

*Resolved,* That a committee of three be appointed to prepare names to fill any vacancies that may exist in the offices of the Association. Drs. Rockwell, Benedict, and Kirkbride, were appointed the committee.

Dr. Rockwell, from the committee to fill vacancies in the offices of the Association, nominated Dr. Luther V. Bell, as Vice President, in place of Dr. A. Brigham, (deceased,) which nomination was confirmed, and Dr. Bell duly elected Vice President of the Association.

An invitation from Drs. Cutter and Howe to visit their institution at Pepperill, Mass., was read, accepted, and referred to the business committee.

Dr. Stedman tendered to the members of the Association, in behalf of the Boston Society for Medical Improvement, an invitation to visit their cabinet, also, one to visit the museum of the Medical College of Harvard University, which were accepted.

Dr. Jarvis tendered invitations to the members, in behalf of the Boston Museum of Natural History, the Boston Atheneum, and the Perkins Institution for the Blind, to visit those institutions, which were accepted.

Dr. Rockwell read a paper on the diet and dietetic regulations for the in[illegible]e; which, after discussion by the members generally, was laid upon the ta[illegible]

A letter was received and read from the librarian of the Massachusetts Historical Society, inviting the members of the Association to visit the Society's rooms during their stay in Boston, which was accepted.

Drs. Beck and Wing took seats with the Association as members of the Board of Managers of the New York State Lunatic Asylum.

Dr. Galt read a paper on the organization of Hospitals for the Insane, and Dr. Higgins on the subject of Resident Superintendents of Hospitals for the Insane. Then adjourned to 4 P. M.

### AFTERNOON SESSION.

The Association met agreeably to adjournment.

The papers read by Drs. Galt and Higgins were called up for consideration, and the whole subject was fully discussed by the members generally, after which the reports were laid upon the table.

Dr. Bates read a report from the standing committee on the Medical Treatment of Insanity, which, after discussion, was laid upon the table.

An invitation from the librarian of the Boston Atheneum, for the members to visit the rooms during their stay in the city was read and accepted. On motion of Dr. Bates, adjourned to 9 A. M., to-morrow.

### SECOND DAY.—MORNING SESSION.

The Association met agreeably to adjournment. The minutes of yesterday's proceedings were read and adopted.

Dr. John R. Allen, of the Kentucky Lunatic Asylum, Dr. John Waddell, of the Provincial Lunatic Asylum at St. Johns, New Brunswick, and

Dr. James Douglass, of the Quebec (Canada) Lunatic Asylum, appeared and took their seats as members of the Association.

Charles Edward Cook, and Otis Clapp, Esqrs., also took seats with the Association as members of the Board of Trustees of the Boston Lunatic Hospital. Dr. Kirkbride, from the committee on business, made a partial report, as required by the resolution of yesterday.

Dr. Ray read a report from the standing committee on the Medical Jurisprudence of Insanity, containing a project for a law regulating the legal relations of the insane, and which had been examined by, and received the sanction of, high judicial and legal authority; after the reading of the paper, on motion of Dr. Kirkbride, it was

*Resolved*, That the committee on business be instructed to have provided forthwith for the use of the members, one hundred copies of the foregoing project of a law, and that the same be made the order of the day for the first session of the Association to-morrow morning.

Dr. Bell from the committee on business made a full report on the objects to be attended to by the Association during the day.

Dr. Bell read a paper on the use of narcotics in the treatment of insanity; after a full discussion of the subject by nearly all the members, the paper was laid upon the table.

Dr. Fonerden read a paper on the Modification of the Brain by habits, which, after discussion, was laid upon the table.

On motion of Dr. Kirkbride, adjourned to meet at the Boston Lunatic Hospital, at 4½ o'clock P. M.

### AFTERNOON SESSION.

The Association, after assembling, proceeded, under the guidance of Dr. Stedman and the Board of Trustees, to visit the Lunatic Hospital and other public institutions at South Boston.

After coming to order for business, Dr. Ranney read a paper on Insanity, as it occurs among the pauper emigrants at the Lunatic Asylum on Blackwell's Island, near New York. After discussion, the paper was laid on the table.

A letter was read from Dr. Fremont, informing the Association that a paper, prepared by him, in reference to the past and present condition of the Insane in Canada East, would be presented to, and read before the Association by his colleague, Dr. Douglass.

On motion of Dr. Galt, adjourned to meet at the Tremont House at 9 o'clock, to-morrow morning.

### THIRD DAY.—MORNING SESSION.

The Association met agreeably to adjournment.

The minutes of yesterday's proceedings were read and adopted.

Dr. Kirkbride, on behalf of the business committee, *moved*, that the consideration of Dr. Ray's project of a law for regulating the legal relations of the Insane, which was made the order of the day for this morning, be deferred for the present, owing to the late period at which the printed copies have been placed in the hands of the members, which motion was agreed to.

On motion of Dr. Allen, it was

*Resolved*, That the Hon. Mayor of the city of Boston, be requested to furnish us, for publication, a report of his eloquent address, delivered at

South Boston on last evening; and also, that the President of this Association be requested to furnish, for the same purpose, his appropriate address in reply.

*Resolved,* That the Secretary furnish each of the above named gentlemen with a copy of the preceding resolution.

An invitation to visit the University of Cambridge, and the Observatory, was received, and accepted for 11 o'clock to-morrow.

An invitation from the Mayor and public authorities of the city of Boston, asking the members of the Association to visit the harbor and Bay, and to inspect the public institutions in the vicinity, to-morrow afternoon, was received and accepted.

The Association, on motion of Dr. Bell, resolved to visit the Massachusetts General Hospital, on the invitation of Dr. Hayward, at 3½ o'clock, and the M'Lean Asylum for the Insane, on his own invitation, at 4½ o'clock this afternoon.

Dr. Galt read a paper on the Medico-legal relations of the Insane, the discussion on which, on motion of Dr. Bates, was deferred till the project of a law, prepared by Dr. Ray, shall come up for consideration.

Dr. Worthington read a paper on the use of baths in the treatment of Insanity, which, after discussion, was laid upon the table.

Dr. Kirkbride, from the standing committee on the construction of Hospitals for the Insane, read a report on that subject, which, after discussion, was laid upon the table.

On motion of Dr. Ray, it was

*Resolved,* That the standing committee on the construction of Hospitals for the Insane, be requested, previous to the next meeting of the Association, to prepare a series of resolutions or propositions, affirming the well-ascertained opinions of this body, in reference to the fundamental principles which should regulate the erection and internal arrangements of American Hospitals for the Insane.

Dr. Jarvis commenced reading a paper on the Comparative Frequency, Curability and Mortality of Insanity in the two sexes; after proceeding for some time, on motion of Dr. Bell, the further reading of the paper was deferred till the next session.

On motion of Dr. Allen, adjourned to meet at the M'Lean Asylum, at 4½ o'clock P. M.

### AFTERNOON SESSION.

Having previously visited the Massachusetts General Hospital, the Association met agreeably to adjournment, at the M'Lean Asylum, under the care of Dr. Bell, and guided by whom, they visited and examined the different parts of that excellent institution.

Having come to order for business, Dr. Jarvis concluded the reading of his paper, commenced this morning, which, after discussion, was laid upon the table.

Dr. Bell, after referring to a paper read by him, before the Association last year, relative to a somewhat peculiar form of mental disease, moved that a committee, consisting of Drs. Awl, Kirkbride, and Douglass, be appointed to visit a case of the disease then under his care in the Asylum, and to report the result of their observations, which was agreed to.

The committee having examined the patient, reported, that it was a well marked case of the form of the disease alluded to, and although not often seen in institutions in the interior, is frequently met with in those near large cities, where cases manifesting much mental disturbance are commonly sent at once to a Hospital for the Insane.

On motion of Dr. Ray, adjourned to meet at the Tremont House, at 8 o'clock to-morrow morning.

FOURTH DAY.—MORNING SESSION.

The Association met agreeably to adjournment.

The minutes of yesterday's proceedings were read and adopted.

Dr. Bell, from the committee on business, made the usual report as to the proceedings of the day.

Dr. Douglass read a paper prepared by his colleague, Dr. Fremont, on the past and present condition of the Insane in Canada East. After discussion, the paper was laid upon the table.

Dr. Galt read a paper on Water Closets, which, after discussion, was laid upon the table. The Association then proceeded to the consideration of the project of a law regulating the legal relations of the Insane, and after a full discussion the further consideration of the subject was postponed until the next session.

On motion of Dr. Bates, adjourned to meet at 9 P. M.

EVENING SESSION.

After visiting the University of Cambridge, and the Observatory, the Association passed the afternoon as the guests of the Corporate authorities of the city of Boston, in an excursion down the Harbor and Bay, in examining the public institutions in that vicinity, and in partaking of the sumptuous hospitality provided on the occasion, and then met for the transaction of business, agreeably to adjournment.

Dr. Bell offered the following resolutions, which were unanimously adopted, viz.:

*Resolved*, That this Association has felt, beyond the power of adequate expression, the profound solemnity, which has been thrown around us, on occasion of its present meeting, by the loss of two of its members so prominent in the history of its organization, as well as in the records of the provision for the Insane in this country, and with still more sensibility, in view of the exalted personal worth, the amiable, cheerful and communicative manners, and pure, self-sacrificing lives of the deceased

*Resolved*, That the deep and general regret which filled the mind of the whole philanthropic community, of an entire section of country and circles where they were best known, uttered in a thousand forms of expression, leaves us in no doubt that their virtues, merits and devotion to great public duties have been appreciated, in a degree commensurate with their just claims, and leaving neither place nor necessity for any long drawn eulogium.

*Resolved*, That notwithstanding the full justice which has been done to the public and private character of our distinguished friends, we still feel that the members of this Association, more intimately and fully acquainted with their peculiar traits of service and sacrifice in our specialty, ought not to be satisfied without a more particular testimonial of our feelings and opinions, as to our deceased brothers; we therefore earnestly and respectfully request that Dr. Chandler would prepare for the next meeting of the

Association, a biographical sketch of the late Dr. Woodward, and that Dr. Nicholls perform the same duty as regards the late Dr. Brigham.

On motion of Dr. Kirkbride, it was

*Resolved,* That Dr. Allen be requested to prepare an obituary notice of our late fellow member, Dr. McNairy, of the Tennessee Hospital for the Insane.

On motion of Dr. Bell, it was

*Resolved,* That the same course be adopted in reference to papers to be read before the Association at its next meeting, as was agreed upon last year.

On motion of Dr. Allen, it was

*Resolved,* That this Association regard with deep interest, the progress of the magnificent project, which has been and continues to be urged by Miss D. L. Dix, on the consideration of Congress, proposing the grant of a portion of the public domain, by the Federal Government, the proceeds of which are to be devoted to the endowment of the public charities throughout the country, and that it meets with our unqualified sanction.

The subject of a project for a law regulating the legal relations of the Insane, being again under consideration, on motion of Dr. Bell, it was

*Resolved,* That the same be re-committed, and that the committee report to the next annual meeting.

On motion of Dr. Allen, it was

*Resolved,* That a committee be appointed to prepare resolutions of thanks to the various public bodies and institutions, official and private citizens, to whom the members of the Association have been indebted for so much of the pleasure of their very gratifying visit to Boston. Drs. Allen, Kirkbride, and Benedict, were appointed the committee.

Dr. Kirkbride tendered to the Association an invitation to hold its next meeting in the city of Philadelphia, when, on motion of Dr. Bell, it was

*Resolved,* That when the Association adjourns, it will adjourn to meet in the city of Philadelphia, on the third Monday of May, 1851, at 10 o'clock, A. M.

On motion of Dr. Bates, adjourned to meet at 8 o'clock to-morrow morning.

FIFTH DAY.—MORNING SESSION.

The Association met agreeably to adjournment.

The minutes of yesterday's proceedings were read and adopted.

Dr. Kirkbride offered the following resolution, which was unanimously adopted, viz.:

*Resolved,* That the members of this Association have visited and examined, with great interest and satisfaction, the McLean Asylum for the Insane, under the care of Dr. Bell, and the Boston Lunatic Hospital, under the care of Dr. Stedman, and desire to express to these gentlemen our sincere thanks for their marked courtesy and attention, for their bountiful hospitality, and for their steady and unwearied efforts to promote our comfort and pleasure during our very gratifying visit to the city of Boston.

Dr. Allen, from the committee appointed last evening, reported the following series of resolutions, which were unanimously adopted, viz.:

*Resolved,* That the grateful acknowledgments of this Association be tendered to the Mayor, Common Council, and the citizens of Boston, for the flattering reception we have met at their hands, and their lavish hospitalities which have been tendered to, and enjoyed by us, and for the pleasure afforded us in a general examination of the public institutions under their control.

*Resolved,* That our thanks are due to the Trustees of the public institutions of South Boston, for polite attention and liberal hospitalities during our visit to their institutions, and to the Trustees of the Massachusetts General Hospital, for similar kindness and attention.

*Resolved,* That our thanks are also due to Drs. Hayward and Townsend, Surgeons, and Mr. R. Girdler, Superintendent of the Massachusetts General Hospital; to Messrs. Harris and Sibley, Librarians, and other officers of Harvard University, and to the Messrs. Bond, of the Observatory, for attentions while visiting the institutions under their charge; and to the officers of the Boston Society for Medical Improvement, Boston Museum of Natural History, Massachusetts Historical Society, Boston Atheneum, and Perkins' Institution for the Blind, for invitations to visit their several institutions, and to the Rev. Lewis Dwight for valuable documents and other attentions.

*Resolved,* That our acknowledgments are due to Messrs. Tucker and Parker, the proprietors of the Tremont House, for the ample and elegant accommodations they have afforded us without charge, for the transaction of the business of the Association.

*Resolved,* That the Secretary be directed to furnish his Honor, the Mayor of Boston, with a copy of the preceding resolutions.

On motion of Dr. Allen, it was

*Resolved,* That the thanks of this Association be tendered to the President, for his able and impartial administration of his arduous duties, and to the Secretary, for his efficient discharge of the laborious functions of his office.

The Treasurer reported, that, after paying all the demands against the Association, there remained a balance of twenty-three cents in his hands.

On motion of Dr. Stedman, it was

*Resolved,* That the Secretary be instructed to furnish a copy of the proceedings of the Association, to the Editor of the American Journal of Insanity, and to the editors of the various medical journals in the United States and Canada, for publication in their respective periodicals.

On motion of Dr. Smith it was

*Resolved,* That a committee of three be appointed by the chair, whose duty it shall be to take into consideration the whole subject of publishing, and to report their views to the Association at its next meeting. Drs. Smith, Allen, and Kirkbride were appointed the committee.

On motion of Mr. Benedict, adjourned to meet in the city of Philadelphia, on the third Monday of May, 1851, at 10 o'clock, A. M.

THOMAS S. KIRKBRIDE, Sec'y.

